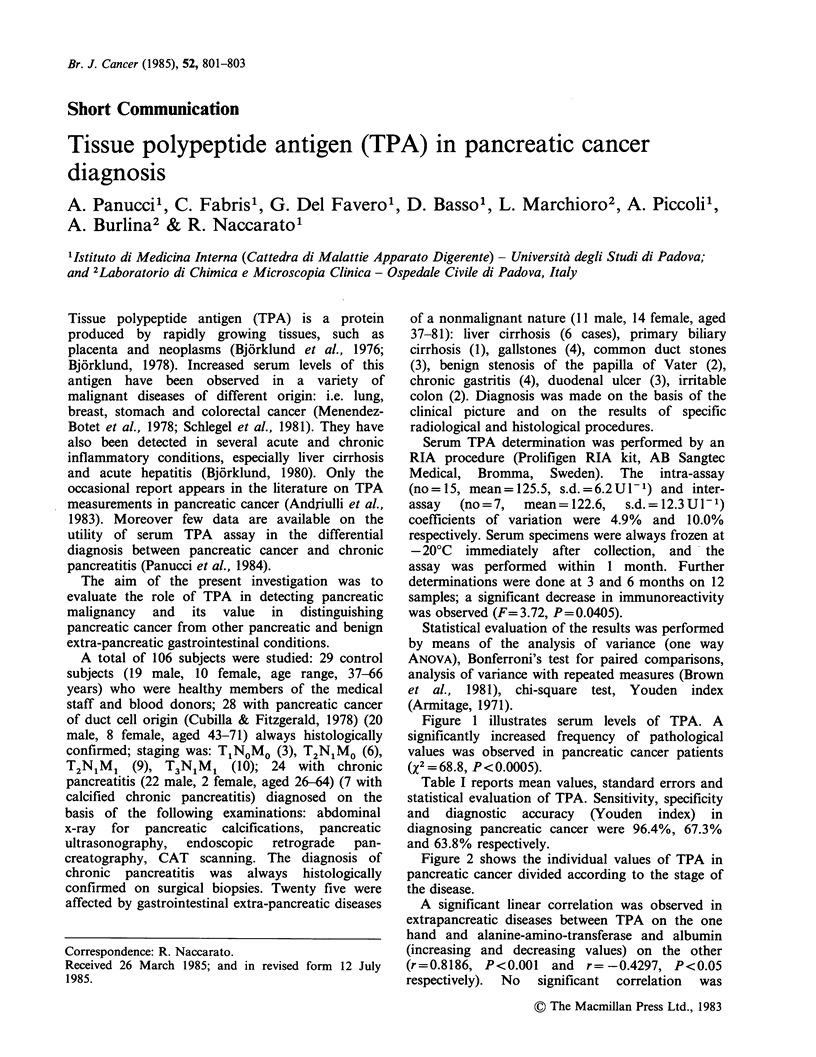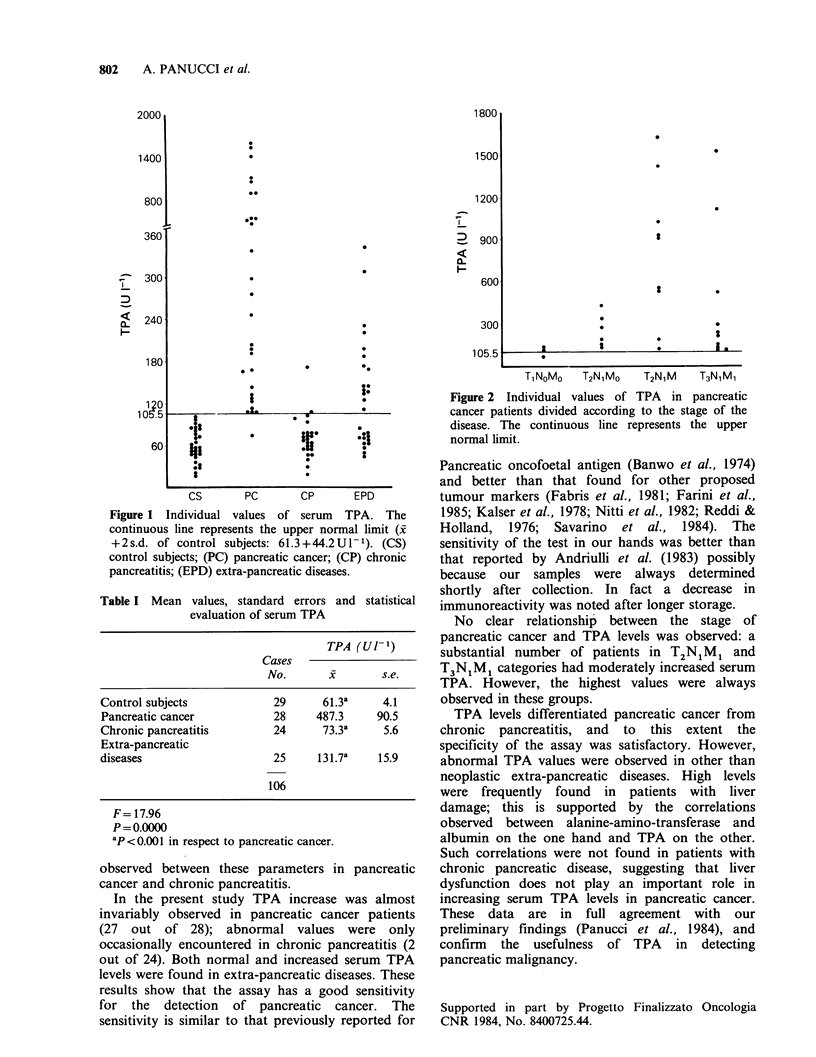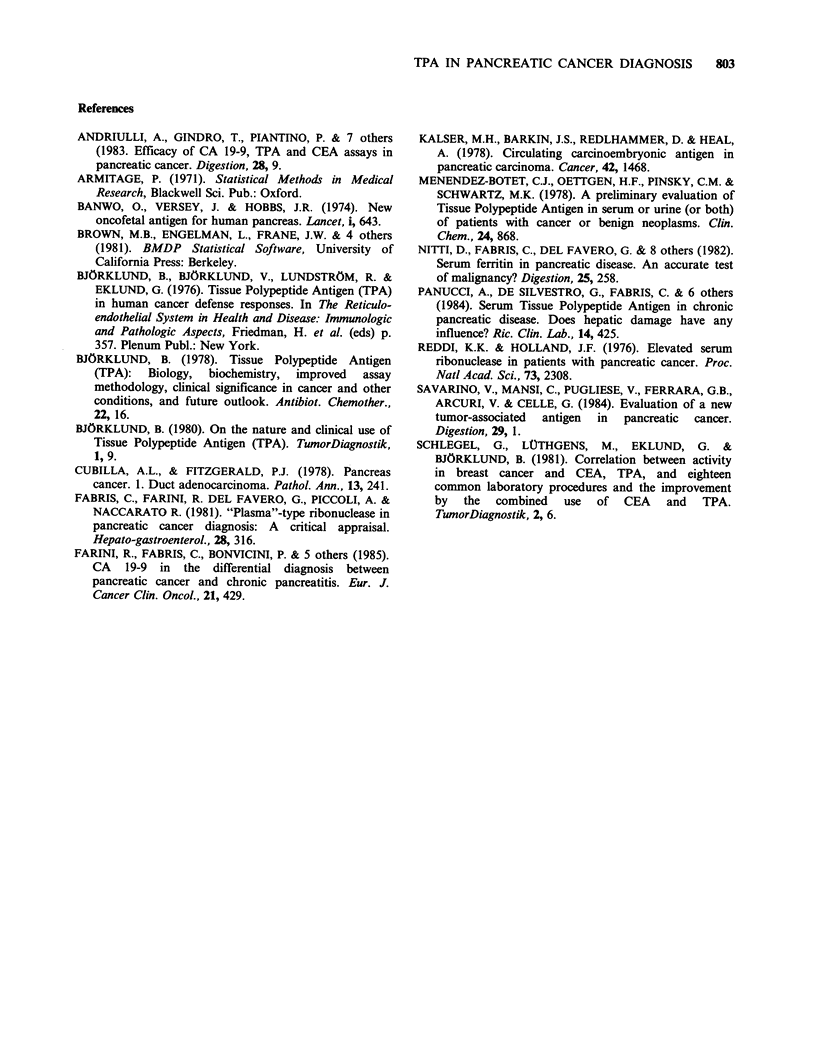# Tissue polypeptide antigen (TPA) in pancreatic cancer diagnosis.

**DOI:** 10.1038/bjc.1985.262

**Published:** 1985-11

**Authors:** A. Panucci, C. Fabris, G. Del Favero, D. Basso, L. Marchioro, A. Piccoli, A. Burlina, R. Naccarato


Br. J. Cancer (1985), 52, 801–803

**Short Communication**

### Tissue polypeptide antigen (TPA) in pancreatic cancer diagnosis

A. Panucci[1], C. Fabris[1], G. Del Favero[1], D. Basso[1], L. Marchioro[2], A. Piccoli[1], A. Burlina[2] & R. Naccarato[1]

[1]*Istituto di Medicina Interna (Cattedra di Malattie Apparato Digerente) – Università degli Studi di Padova; and* [2]*Laboratorio di Chimica e Microscopia Clinica – Ospedale Civile di Padova, Italy*

Tissue polypeptide antigen (TPA) is a protein produced by rapidly growing tissues, such as placenta and neoplasms (Björklund *et al.*, 1976; Björklund, 1978). Increased serum levels of this antigen have been observed in a variety of malignant diseases of different origin: i.e. lung, breast, stomach and colorectal cancer (Menendez-Botet *et al.*, 1978; Schlegel *et al.*, 1981). They have also been detected in several acute and chronic inflammatory conditions, especially liver cirrhosis and acute hepatitis (Björklund, 1980). Only the occasional report appears in the literature on TPA measurements in pancreatic cancer (Andriulli *et al.*, 1983). Moreover few data are available on the utility of serum TPA assay in the differential diagnosis between pancreatic cancer and chronic pancreatitis (Panucci *et al.*, 1984).

The aim of the present investigation was to evaluate the role of TPA in detecting pancreatic malignancy and its value in distinguishing pancreatic cancer from other pancreatic and benign extra-pancreatic gastrointestinal conditions.

A total of 106 subjects were studied: 29 control subjects (19 male, 10 female, age range, 37–66 years) who were healthy members of the medical staff and blood donors; 28 with pancreatic cancer of duct cell origin (Cubilla & Fitzgerald, 1978) (20 male, 8 female, aged 43–71) always histologically confirmed; staging was: $T_1N_0M_0$ (3), $T_2N_1M_0$ (6), $T_2N_1M_1$ (9), $T_3N_1M_1$ (10); 24 with chronic pancreatitis (22 male, 2 female, aged 26–64) (7 with calcified chronic pancreatitis) diagnosed on the basis of the following examinations: abdominal x-ray for pancreatic calcifications, pancreatic ultrasonography, endoscopic retrograde pancreatography, CAT scanning. The diagnosis of chronic pancreatitis was always histologically confirmed on surgical biopsies. Twenty five were affected by gastrointestinal extra-pancreatic diseases of a nonmalignant nature (11 male, 14 female, aged 37–81): liver cirrhosis (6 cases), primary biliary cirrhosis (1), gallstones (4), common duct stones (3), benign stenosis of the papilla of Vater (2), chronic gastritis (4), duodenal ulcer (3), irritable colon (2). Diagnosis was made on the basis of the clinical picture and on the results of specific radiological and histological procedures.

Serum TPA determination was performed by an RIA procedure (Prolifigen RIA kit, AB Sangtec Medical, Bromma, Sweden). The intra-assay (no = 15, mean = 125.5, s.d. = $6.2\,U\,l^{-1}$) and inter-assay (no = 7, mean = 122.6, s.d. = $12.3\,U\,l^{-1}$) coefficients of variation were 4.9% and 10.0% respectively. Serum specimens were always frozen at −20°C immediately after collection, and the assay was performed within 1 month. Further determinations were done at 3 and 6 months on 12 samples; a significant decrease in immunoreactivity was observed ($F = 3.72$, $P = 0.0405$).

Statistical evaluation of the results was performed by means of the analysis of variance (one way ANOVA), Bonferroni's test for paired comparisons, analysis of variance with repeated measures (Brown *et al.*, 1981), chi-square test, Youden index (Armitage, 1971).

Figure 1 illustrates serum levels of TPA. A significantly increased frequency of pathological values was observed in pancreatic cancer patients ($\chi^2 = 68.8$, $P < 0.0005$).

Table I reports mean values, standard errors and statistical evaluation of TPA. Sensitivity, specificity and diagnostic accuracy (Youden index) in diagnosing pancreatic cancer were 96.4%, 67.3% and 63.8% respectively.

Figure 2 shows the individual values of TPA in pancreatic cancer divided according to the stage of the disease.

A significant linear correlation was observed in extrapancreatic diseases between TPA on the one hand and alanine-amino-transferase and albumin (increasing and decreasing values) on the other ($r = 0.8186$, $P < 0.001$ and $r = -0.4297$, $P < 0.05$ respectively). No significant correlation was

Correspondence: R. Naccarato.
Received 26 March 1985; and in revised form 12 July 1985.

© The Macmillan Press Ltd., 1983

**Figure 1** Individual values of serum TPA. The continuous line represents the upper normal limit ($\bar{x}$ $+2$s.d. of control subjects: $61.3+44.2\,\mathrm{U\,l^{-1}}$). (CS) control subjects; (PC) pancreatic cancer; (CP) chronic pancreatitis; (EPD) extra-pancreatic diseases.

**Table I** Mean values, standard errors and statistical evaluation of serum TPA

| | Cases No. | TPA ($U\,l^{-1}$) $\bar{x}$ | s.e. |
|---|---|---|---|
| Control subjects | 29 | 61.3[a] | 4.1 |
| Pancreatic cancer | 28 | 487.3 | 90.5 |
| Chronic pancreatitis | 24 | 73.3[a] | 5.6 |
| Extra-pancreatic diseases | 25 | 131.7[a] | 15.9 |
| | 106 | | |

$F=17.96$

$P=0.0000$

[a]$P<0.001$ in respect to pancreatic cancer.

observed between these parameters in pancreatic cancer and chronic pancreatitis.

In the present study TPA increase was almost invariably observed in pancreatic cancer patients (27 out of 28); abnormal values were only occasionally encountered in chronic pancreatitis (2 out of 24). Both normal and increased serum TPA levels were found in extra-pancreatic diseases. These results show that the assay has a good sensitivity for the detection of pancreatic cancer. The sensitivity is similar to that previously reported for

**Figure 2** Individual values of TPA in pancreatic cancer patients divided according to the stage of the disease. The continuous line represents the upper normal limit.

Pancreatic oncofoetal antigen (Banwo et al., 1974) and better than that found for other proposed tumour markers (Fabris et al., 1981; Farini et al., 1985; Kalser et al., 1978; Nitti et al., 1982; Reddi & Holland, 1976; Savarino et al., 1984). The sensitivity of the test in our hands was better than that reported by Andriulli et al. (1983) possibly because our samples were always determined shortly after collection. In fact a decrease in immunoreactivity was noted after longer storage.

No clear relationship between the stage of pancreatic cancer and TPA levels was observed: a substantial number of patients in $T_2N_1M_1$ and $T_3N_1M_1$ categories had moderately increased serum TPA. However, the highest values were always observed in these groups.

TPA levels differentiated pancreatic cancer from chronic pancreatitis, and to this extent the specificity of the assay was satisfactory. However, abnormal TPA values were observed in other than neoplastic extra-pancreatic diseases. High levels were frequently found in patients with liver damage; this is supported by the correlations observed between alanine-amino-transferase and albumin on the one hand and TPA on the other. Such correlations were not found in patients with chronic pancreatic disease, suggesting that liver dysfunction does not play an important role in increasing serum TPA levels in pancreatic cancer. These data are in full agreement with our preliminary findings (Panucci et al., 1984), and confirm the usefulness of TPA in detecting pancreatic malignancy.

Supported in part by Progetto Finalizzato Oncologia CNR 1984, No. 8400725.44.

### References

ANDRIULLI, A., GINDRO, T., PIANTINO, P. & 7 others (1983. Efficacy of CA 19-9, TPA and CEA assays in pancreatic cancer. *Digestion*, **28**, 9.

ARMITAGE, P. (1971). *Statistical Methods in Medical Research*, Blackwell Sci. Pub.: Oxford.

BANWO, O., VERSEY, J. & HOBBS, J.R. (1974). New oncofetal antigen for human pancreas. *Lancet*, **i**, 643.

BROWN, M.B., ENGELMAN, L., FRANE, J.W. & 4 others (1981). *BMDP Statistical Software*, University of California Press: Berkeley.

BJÖRKLUND, B., BJÖRKLUND, V., LUNDSTRÖM, R. & EKLUND, G. (1976). Tissue Polypeptide Antigen (TPA) in human cancer defense responses. In *The Reticulo-endothelial System in Health and Disease: Immunologic and Pathologic Aspects*, Friedman, H. *et al.* (eds) p. 357. Plenum Publ.: New York.

BJÖRKLUND, B. (1978). Tissue Polypeptide Antigen (TPA): Biology, biochemistry, improved assay methodology, clinical significance in cancer and other conditions, and future outlook. *Antibiot. Chemother.*, **22**, 16.

BJÖRKLUND, B. (1980). On the nature and clinical use of Tissue Polypeptide Antigen (TPA). *TumorDiagnostik*, **1**, 9.

CUBILLA, A.L., & FITZGERALD, P.J. (1978). Pancreas cancer. 1. Duct adenocarcinoma. *Pathol. Ann.*, **13**, 241.

FABRIS, C., FARINI, R. DEL FAVERO, G., PICCOLI, A. & NACCARATO R. (1981). "Plasma"-type ribonuclease in pancreatic cancer diagnosis: A critical appraisal. *Hepato-gastroenterol.*, **28**, 316.

FARINI, R., FABRIS, C., BONVICINI, P. & 5 others (1985). CA 19-9 in the differential diagnosis between pancreatic cancer and chronic pancreatitis. *Eur. J. Cancer Clin. Oncol.*, **21**, 429.

KALSER, M.H., BARKIN, J.S., REDLHAMMER, D. & HEAL, A. (1978). Circulating carcinoembryonic antigen in pancreatic carcinoma. *Cancer*, **42**, 1468.

MENENDEZ-BOTET, C.J., OETTGEN, H.F., PINSKY, C.M. & SCHWARTZ, M.K. (1978). A preliminary evaluation of Tissue Polypeptide Antigen in serum or urine (or both) of patients with cancer or benign neoplasms. *Clin. Chem.*, **24**, 868.

NITTI, D., FABRIS, C., DEL FAVERO, G. & 8 others (1982). Serum ferritin in pancreatic disease. An accurate test of malignancy? *Digestion*, **25**, 258.

PANUCCI, A., DE SILVESTRO, G., FABRIS, C. & 6 others (1984). Serum Tissue Polypeptide Antigen in chronic pancreatic disease. Does hepatic damage have any influence? *Ric. Clin. Lab.*, **14**, 425.

REDDI, K.K. & HOLLAND, J.F. (1976). Elevated serum ribonuclease in patients with pancreatic cancer. *Proc. Natl Acad. Sci.*, **73**, 2308.

SAVARINO, V., MANSI, C., PUGLIESE, V., FERRARA, G.B., ARCURI, V. & CELLE, G. (1984). Evaluation of a new tumor-associated antigen in pancreatic cancer. *Digestion*, **29**, 1.

SCHLEGEL, G., LÜTHGENS, M., EKLUND, G. & BJÖRKLUND, B. (1981). Correlation between activity in breast cancer and CEA, TPA, and eighteen common laboratory procedures and the improvement by the combined use of CEA and TPA. *TumorDiagnostik*, **2**, 6.